# Post-Cardiac Injury Syndrome: A Paradigm Shift in Diagnosis and Management

**DOI:** 10.1016/j.jacadv.2025.102166

**Published:** 2025-10-22

**Authors:** Nicholas M. Platek, Ian Persits, Mohamed Khayata, Osamah Z. Badwan, Joseph El Roumi, Allan L. Klein

**Affiliations:** a Department of Internal Medicine, Cleveland Clinic, Cleveland, Ohio, USA; b Cardiovascular Medicine Department, Cleveland Clinic, Cleveland, Ohio, USA; c Center for the Diagnosis and Treatment of Pericardial Diseases, Section of Cardiovascular Imaging, Department of Cardiovascular Medicine Heart Vascular and Thoracic Institute, Cleveland Clinic, Cleveland, Ohio, USA

**Keywords:** multimodality imaging, pericardial disease, pericarditis, post-cardiac injury syndrome

## Abstract

Post-cardiac injury syndrome (PCIS) refers to a group of inflammatory pericardial syndromes that arise after cardiac injury, encompassing post-myocardial infarction syndrome, post-pericardiotomy syndrome, and post-traumatic pericarditis. PCIS has evolved significantly over recent years, transitioning from a rare post-myocardial infarction syndrome to a more common phenomenon stemming from the rise of both surgical and transcatheter cardiac procedures. The increasing prevalence of these procedures makes understanding and managing PCIS an important clinical consideration. Additionally, in rare instances of post-MI pericarditis, balancing the reduction of inflammation while maintaining structural cardiac integrity for healing presents unique therapeutic challenges. In this review, we aim to provide a comprehensive overview of PCIS, focusing on its most common manifestations, key imaging modalities for diagnosis, and novel therapeutic strategies.

## Highlights

• Cardiac procedures have increased the prevalence and clinical relevance of PCIS.
• Multimodality imaging, especially cardiac magnetic resonance, improves PCIS diagnosis and guides personalized treatment strategies.
• IL-1–targeted therapies are emerging options for recurrent or refractory PCIS.
• Standardized diagnostic criteria and equitable access to therapies are key future directions.

Post-cardiac injury syndrome (PCIS) refers to a group of inflammatory pericardial syndromes that arise after cardiac injury, encompassing: 1) post-myocardial infarction syndrome (PMIS); 2) post-pericardiotomy syndrome (PPS); and 3) post-traumatic pericarditis. Despite distinct triggers, all share an underlying autoinflammatory process.^1^

While PMIS, the first of the class to be described, has become rare, the increasing prevalence of cardiac surgeries and minimally invasive procedures has resulted in a paradigm shift of rising rates of PPS and post-traumatic pericarditis. However, diagnosing PCIS remains challenging, requiring careful assessment of the inciting event, clinical findings, laboratory markers, and multimodality imaging. Treatment, especially for recurrent cases, must be individualized based on patients’ circumstances and mechanisms of pericardial inflammation.

As such, PCIS remains a clinically significant condition that requires careful consideration. This review explores the latest diagnostic and treatment strategies, covering pathophysiology, prevention, and approaches from initial presentation to refractory cases. We highlight multimodality imaging, emerging therapies, including interleukin (IL)-1 inhibitors, and discuss specific etiologies requiring a targeted treatment approach.

## Epidemiology

### Post-myocardial infarction syndrome

First described by Dressler in 1956, PMIS was the earliest recognized form of PCIS.^2^ While both early and late post-MI pericarditis bear Dressler's name, the latter, occurring weeks to months after infarction, is most often referred to in the context of PCIS. In contrast, early post-MI pericarditis occurs within days, typically following a transmural infarction or late-presenting MI complicated by ventricular free wall rupture.

With the dawn of the reperfusion era, PMIS has become the rarest PCIS subtype. Prereperfusion era studies reported an incidence of 1% to 5%, but modern studies report rates below 1%, with both early and late forms occurring in <5% and <1% of cases, respectively.^3^, ^4^, ^5^, ^6^, ^7^, ^8^ This decline is largely attributed to early revascularization which limits myocardial injury, as well as the potential immunomodulatory effects of standard post-MI therapies.

### Post-pericardiotomy syndrome

The increasing volume of cardiac surgeries and minimally invasive procedures has led to a rising incidence of PPS and post-traumatic pericarditis. PPS, characterized by pericarditis developing weeks to months after cardiac surgery, was initially termed post-commissurotomy syndrome, as it was first described in patients undergoing mitral commissurotomy. As more cardiac procedures were implicated, the term post-cardiotomy syndrome emerged, eventually evolving into the current PPS, which encompasses any cardiac surgery involving pericardial manipulation.

Retrospective studies estimate PPS incidence at 10% to 40%, while more recent prospective studies report rates of 21% to 29% in post-cardiac surgery patients.^9^, ^10^, ^11^, ^12^, ^13^, ^14^ However, the incidence varies by procedure type. A large study of 28,761 patients found ascending aortic surgery to carry the highest PPS risk, followed by aortic valve replacement, mitral valve replacement, and coronary artery bypass grafting likely due to the extent of direct pericardial trauma, pericardial bleeding, and individual predisposition.^15^ Additionally, a smaller study of 360 patients suggested that undergoing multiple surgical procedures in a single operation increased PPS risk.^16^ While there is a paucity of larger studies implicating minimally invasive cardiac procedures, there are several noted case reports of PPS following transcatheter aortic valve implantation, transcatheter edge-to-edge mitral repair, and endocardial lead fixation.^17^, ^18^, ^19^, ^20^, ^21^

### Post-traumatic pericarditis

Iatrogenic pericarditis is a notable complication following various cardiac interventions, with an incidence ranging from 0.5% to 5%, depending on the procedure.^22^ Percutaneous coronary interventions (PCI), pacemaker lead insertions, radiofrequency ablation, and pulmonary arterial catheterization have all been implicated in myocardial injury leading to post-traumatic pericarditis, as documented in multiple case reports.^22^, ^23^, ^24^, ^25^

The National Cardiovascular Data Registry AFib Ablation Registry, the largest prospective database on atrial fibrillation ablation, highlights a rise in procedural complications alongside increasing ablation volumes. Although pericarditis incidence was not explicitly reported, 0.26% of patients developed pericardial effusion (PEff) with cardiac tamponade, while 0.44% required pericardial intervention, both potential precursors to post-traumatic pericarditis.^26^ A retrospective cohort study of 2,215 patients undergoing atrial fibrillation ablation found that 10.2% developed suspected acute pericarditis, with treatment strategies including colchicine (65.9%), prednisone (29.2%), and high-dose ibuprofen (19.0%).^27^

Given the rise in such cardiac interventions, so does the ongoing risk of traumatic pericarditis despite advances in procedural techniques. As such, it is imperative for physicians performing these procedures to remain aware of such complications, as prompt recognition and appropriate management of pericarditis remain essential to mitigating long-term sequalae.

## Pathophysiology

While the precise mechanisms underlying PCIS remain unclear, it is evident that the immune system drives the inflammatory response (Central Illustration). Exposure to endogenous intracellular antigens, whether from myocardial necrosis (post-MI), iatrogenic trauma (post-pericardiotomy), or blunt cardiac injury, triggers an innate immune reaction.^28^, ^29^, ^30^, ^31^, ^32^

**Central Illustration:**
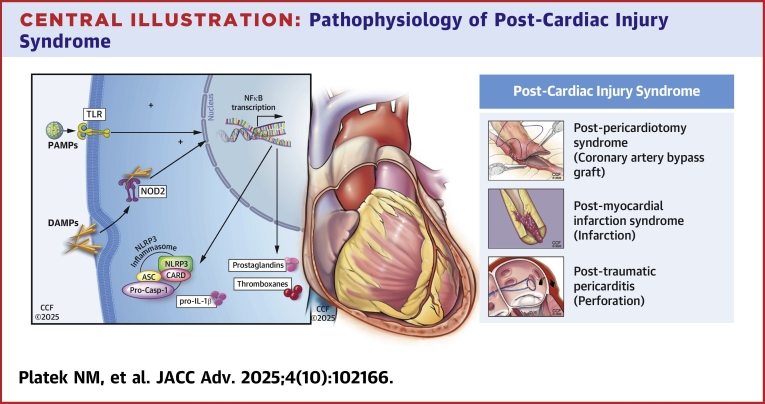
**Pathophysiology of Post-Cardiac Injury Syndrome** Pericardial injury leads to the release of damage- and pathogen-associated molecular patterns (DAMPs/PAMPs), activating nuclear factor kappa-light-chain enhancer of activated B cells (NF-κB signaling), which upregulates inflammatory mediators and cytokines essential for inflammasome assembly. The inflammasome then facilitates the release of IL-1β and IL-18, amplifying the inflammatory cascade. Additionally, NF-κB promotes phospholipase A2 synthesis, driving the arachidonic acid pathway and producing prostaglandins and thromboxanes. IL-1α and IL-1β, acting through the IL-1 receptor (IL-1R), further propagate pericardial inflammation, with IL-1α serving as an early alarmin and IL-1β enhancing the immune response.^25^^,^^33^, ^34^, ^35^ Abbreviation as in Figure 6.

Pericardial injury leads to the release of damage- and pathogen-associated molecular patterns, activating nuclear factor kappa-light-chain enhancer of activated B cells (NF-κB signaling), which upregulates inflammatory mediators and cytokines essential for inflammasome assembly. The inflammasome then facilitates the release of IL-1β and IL-18, amplifying the inflammatory cascade. Additionally, NF-κB promotes phospholipase A2 synthesis, driving the arachidonic acid pathway and producing prostaglandins and thromboxanes. IL-1α and IL-1β, acting through the IL-1 receptor (IL-1R), further propagate pericardial inflammation, with IL-1α serving as an early alarmin and IL-1β enhancing the immune response.^25^^,^^33^, ^34^, ^35^

Emerging research also suggests a role for immune complex deposition, with several studies detecting antimyocardial antibodies in patients with PPS, further implicating an autoimmune component in PCIS pathogenesis.^27^^,^^31^^,^^36^

## Clinical presentation/diagnostic criteria

The latency period between cardiac injury and symptom onset in PCIS varies, making diagnosis challenging. The most common symptoms include pleuritic chest pain, low-grade fever, dyspnea, and pericardial friction rub, though their presentation can be inconsistent. Electrocardiograms (ECGs) may show diffuse ST-segment elevations and PR depressions, but these findings are relatively uncommon. Inflammatory markers, such as C-reactive protein (CRP) and Westergren sedimentation rate (method used to measure the erythrocyte sedimentation rate), are often elevated.

The 2015 European Society of Cardiology guidelines define pericarditis based on the presence of at least 2 of the following criteria: 1) chest pain (>85 to 90% of cases)—typically sharp, pleuritic, and improved by sitting up and leaning forward; 2) pericardial friction rub (≤33% of cases)—a superficial, scratchy sound best heard with the diaphragm of the stethoscope over the left sternal border; 3) ECG changes (up to 60% of cases)—new widespread ST-segment elevation or PR depression in the acute phase; and 4) PEff (up to 60% of cases, generally mild).^32^ The 2025 European Society of Cardiology Guidelines for the Management of Myocarditis and Pericarditis expand on these criteria by clarifying that a definite clinical diagnosis of pericarditis requires a compatible presentation and more than one additional criterion. They reaffirm chest pain as the most common presenting feature and highlight the importance of inflammatory markers such as CRP, erythrocyte sedimentation rate, and neutrophilic leukocytosis as supportive criteria. Furthermore, the guidelines place greater emphasis on advanced imaging, particularly CMR, for tissue characterization, detection of pericardial edema, and late gadolinium enhancement, which can strengthen the diagnosis in equivocal cases.^37^ Similarly, the new ACC 2025 Concise Clinical Guidance: An ACC Expert Consensus Statement on the Diagnosis and Management of Pericarditis also provides similar new definitions of pericarditis dividing the patients into definite, possible, and unlikely based on the classic pericarditis chest pain and number of positive criteria.^38^ To diagnose a patient with PCIS, 2 of the following 5 proposed diagnostic criteria need to be fulfilled: 1) fever without alternative causes; 2) pericarditic or pleuritic chest pain; 3) pericardial or pleural rubs; 4) evidence of PEff; and/or 5) pleural effusion with elevated CRP.^37^ Current diagnostic criteria for PCIS have not been prospectively validated and rely on extrapolations from broader pericarditis guidelines. This can lead to diagnostic uncertainty, particularly in postoperative patients where overlapping inflammatory responses may confound assessment. Additionally, there is a paucity of data on how these criteria perform in diverse populations and across different procedural contexts. Multicenter studies with standardized imaging and biomarker thresholds are needed to refine the diagnostic framework and ensure it remains applicable across a broad clinical spectrum.

Distinguishing PCIS from other causes of postoperative pericardial inflammation remains challenging, particularly in the early postoperative period when symptoms such as fever, chest pain, and effusions may overlap with infectious or mechanical complications. Postoperative PEff can result from surgical trauma, bleeding, or infection, rather than an autoinflammatory process. While elevated inflammatory markers and symptom timing are helpful, they are not specific. Advanced imaging can improve diagnostic confidence. Features such as pericardial edema and pericardial late gadolinium enhancement (LGE) on CMR may suggest active inflammation consistent with PCIS, whereas abscesses or loculated effusions with signs of systemic infection may suggest alternative etiologies. Further research is needed to validate imaging-based criteria and biomarkers that reliably differentiate PCIS from other postoperative conditions.

## Multimodality imaging

**Figure 1:**
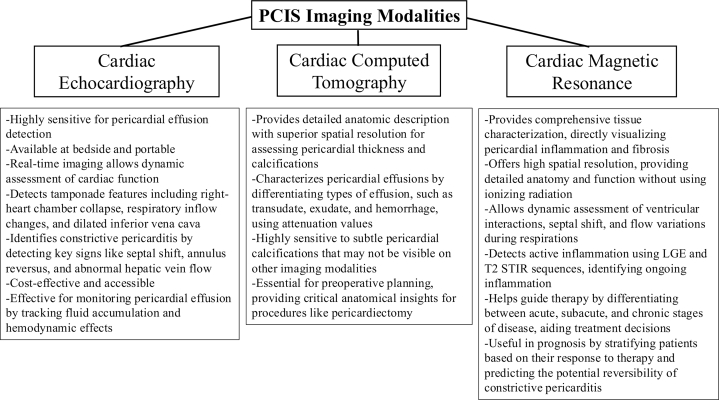
**PCIS Imaging Modalities** Overview of the strengths of echocardiography, CCT, and CMR in PCIS evaluation and treatment. CCT = cardiac computed tomography; IVC = inferior vena cava; LGE = late gadolinium enhancement; PCIS = post-cardiac injury syndrome; T2-STIR = T2-Weighted Short-TI Inversion Recovery.

Advanced imaging plays a crucial role in quantifying inflammation and localizing pericardial involvement, offering greater diagnostic clarity in PCIS. Each modality provides unique advantages, complementing clinical assessment and inflammatory markers in guiding management. Figure 1 provides an overview of the strengths of the three primary noninvasive modalities used to evaluate PCIS: echocardiography, cardiac computed tomography, and cardiac magnetic resonance (CMR).

### Echocardiography

As the first-line imaging modality, echocardiography is widely used due to its low cost, safety, and availability. It is highly effective in detecting and characterizing PEffs while providing hemodynamic insights. Notably, in post-MI PCIS, effusions >10 mm are associated with a higher risk of cardiac tamponade or left ventricular free wall rupture.^39^ Additionally, echocardiography helps monitor effusion resolution and detect constrictive physiology, but it must be correlated with clinical findings and inflammatory markers for a comprehensive assessment.^40^

**Figure 2:**
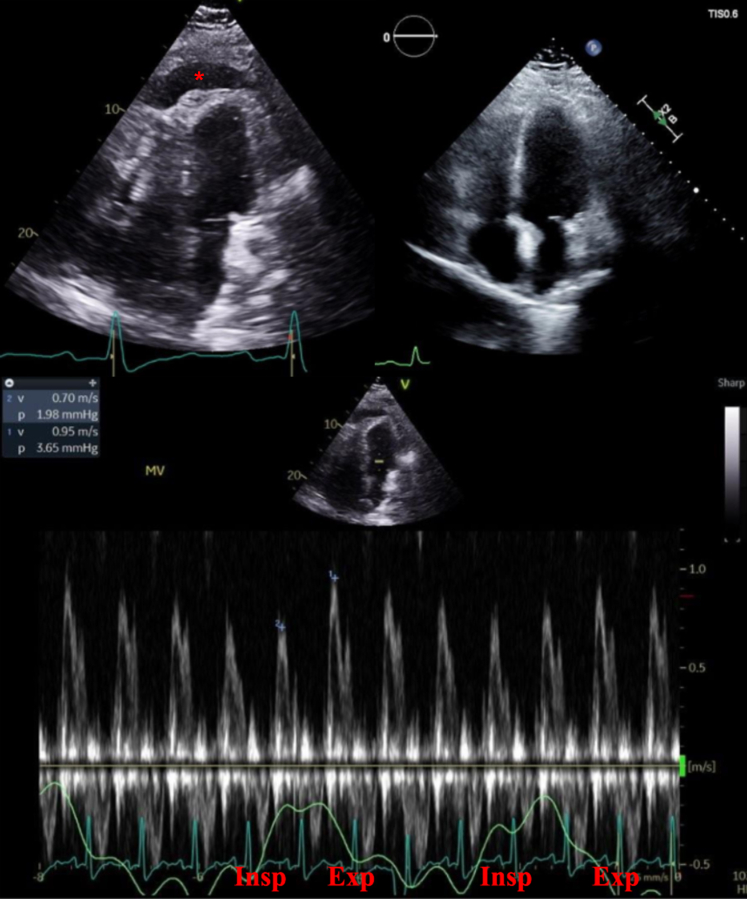
Case Presentation #1 A 50-year-old male who underwent a 4-vessel coronary artery bypass grafting which was complicated by Dressler syndrome. The upper left echo image shows a large PEff on a postoperative transthoracic echocardiogram apical 4-chamber view (red asterisk marks the PEff). The upper right echo image reveals the same TTE view with resolution of that PEff after treatment with aspirin and colchicine. The bottom image again shows the PEff on echocardiogram along with respiratory variation across the mitral valve on Doppler inflow pattern. Exp = expiration; Insp = inspiration; PEff = pericardial effusion; TTE = transthoracic echocardiogram.

Figure 2 shows an echocardiogram of a patient status post coronary artery bypass grafting diagnosed with Dressler syndrome. Initial imaging revealed a large PEff along with respiratory variation across the mitral valve indicating dissociation between intrathoracic and intracardiac pressure and interventricular dependence. This patient's pericarditis resolved after treatment with aspirin and colchicine.

### Computerized tomography

Cardiac computed tomography imaging offers detailed visualization of pericardial thickness and aids in differentiating various types of pericarditis. It is particularly useful in detecting pericardial and pleural effusions, identifying postoperative mediastinal changes, and assessing active pericardial inflammation using iodinated contrast.^41^ Pericardial fluid composition can be evaluated through Hounsfield unit (HU) attenuation. More specifically, transudate is usually <10 HU, exudate 20 to 60 HU, and hemorrhage >60 HU.^42^ However, CT has limitations in patients with renal dysfunction, arrhythmias, or recent stent placement, where blooming artifacts may distort imaging. In such cases, CMR becomes the preferred alternative.^43^

### Cardiac magnetic resonance

**Figure 3:**
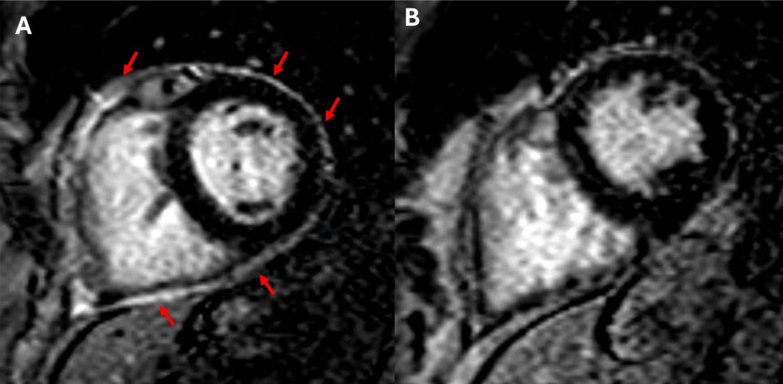
Case Presentation #2 A 66-year-old female diagnosed with pericarditis post pacemaker implantation. Figure A is a short-axis slice of cardiac magnetic resonance imaging showing severe late gadolinium enhancement (arrows). Figure B is repeat imaging in the same patient 1 year later with improved inflammation after completion of treatment with NSAIDs and colchicine. NSAIDs = nonsteroidal anti-inflammatory drug.

**Figure 4:**
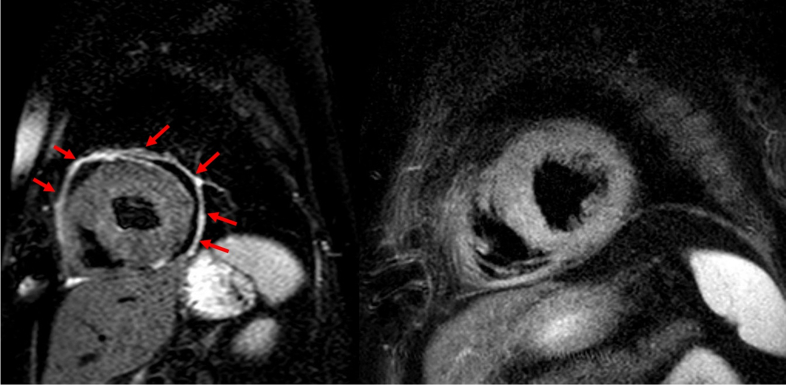
Case Presentation #3 A 53-year-old female with a history of hypertrophic cardiomyopathy who developed recurrent pericarditis following pericardiotomy (septal myectomy). The image on the left is a short-axis slice of cardiac magnetic resonance imaging showing late gadolinium enhancement suggestive of inflammation (arrows). The image on the right is repeat imaging in the same patient 1 year later with improved enhancement after completion of treatment with NSAIDs, colchicine, and prednisone. Abbreviation as in Figure 3.

**Figure 5:**
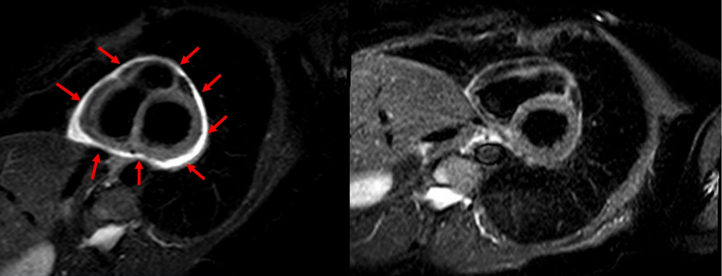
Case Presentation #4 A 66-year-old female who developed recurrent pericarditis after a coronary perforation during a percutaneous coronary intervention of her left anterior descending artery. The image on the left is a short-axis slice of cardiac magnetic resonance imaging showing late gadolinium enhancement, edema, and pericardial thickening (arrows). The image on the right is repeat imaging of the same patient 1 year later after completion of treatment with aspirin, colchicine, and prednisone noting significant improvement.

CMR provides unparalleled resolution in detecting and quantifying pericardial fluid, inflammation, and fibrosis, making it the most comprehensive imaging modality for pericardial disease. Using LGE, CMR can identify active inflammation with a sensitivity comparable to histopathology.^44^ Additional sequences, such as T1/T2 mapping and postcontrast imaging, help differentiate acute, subacute, and chronic pericardial inflammation.^40^ Figure 3, Figure 4, Figure 5 highlight 3 different cases of pericarditis, one post pacemaker implantation, a second post septal myectomy of a patient with hypertrophic obstructive cardiomyopathy, and the third after a coronary perforation during a PCI of her left anterior descending artery. In these images, you can appreciate how CMR can identify differing degrees of pericardial thickening, edema, and inflammation using Short-TI Inversion Recovery-T2 weighted imaging, myocardial fibrosis using LGE sequences, and PEffs. CMR enables more precise delineation of inflammation originating specifically from the pericardium, which is critical for accurate diagnosis and treatment planning.

Despite its diagnostic superiority, CMR has its limitations such as high cost and limited availability outside of major academic medical centers. Moreover, it is contraindicated in patients with non-magnetic resonance imaging-compatible cardiac devices. Nonetheless, its ability to precisely localize and characterize pericardial inflammation makes CMR an invaluable tool for accurate diagnosis and tailored management of PCIS.

## Management strategies in post-cardiac injury syndrome

The treatment of PCIS follows a standard anti-inflammatory approach, utilizing nonsteroidal anti-inflammatory drugs (NSAIDs), colchicine, and exercise restriction to control acute pericardial inflammation.^45^ Corticosteroids are reserved for cases where first-line therapy is contraindicated or ineffective. While this strategy is effective for many patients, PCIS presents unique challenges, particularly in those with renal dysfunction, gastrointestinal disorders, high bleeding risks, diabetes, or infections, necessitating individualized management.

A meta-analysis of 10 randomized controlled trials (n = 1,981) demonstrated that colchicine significantly reduces pericarditis recurrence and decreases PPS incidence (relative risk: 0.57).^46^ However, its use is associated with an increased rate of adverse effects (relative risk: 1.42), predominantly gastrointestinal intolerance. Building on these findings, the Colchicine for Prevention of Postpericardiotomy Syndrome and Postoperative Atrial Fibrillation (COPPS-2) randomized clinical trial showed reduced PPS incidence with preoperative colchicine, while the Colchicine to Prevent Periprocedural Myocardial Injury in Percutaneous Coronary Intervention (COPE-PCI) pilot trial demonstrated reduced myocardial injury when colchicine was given before PCI.^13^^,^^47^ Additionally, prophylactic colchicine before atrial fibrillation ablation lowered the risk of postprocedural pericarditis. These data suggest colchicine may have a broader role in preventing PCIS in high-risk procedural settings.

The use of NSAIDs and steroids after MI remains controversial due to concerns about impaired healing and increased bleeding risk. Given the vulnerability of friable myocardium, treatment must be tailored to the type of MI and underlying pathology. Early post-MI pericarditis is rare in the reperfusion era and generally self-limited, requiring supportive care. However, if symptoms persist, aspirin plus colchicine may be used, while steroids should be avoided. On the other hand, late post-MI pericarditis follows standard empiric therapy, though clinicians must evaluate for residual ischemia using high-sensitivity troponin, ECG, and inflammatory markers.^33^ Timely PCI remains the most critical strategy in reducing post-MI pericarditis incidence.

PEffs are common after cardiac surgery, but management depends on the presence of systemic inflammation. In patients without systemic inflammation, NSAIDs and colchicine should be avoided, as they do not reduce effusion volume or prevent late cardiac tamponade.^48^^,^^49^ Additionally, effusion size is a key determinant: A retrospective study found that a postoperative PEff grade <2 on echocardiography by day 20 had a 100% negative predictive value for late tamponade, suggesting that these cases do not require early intervention.^50^

Despite the rising incidence of PCIS due to the growing number of cardiac interventions, the overall prognosis remains favorable. In PPS following cardiac surgery, recurrence rates are <4%, cardiac tamponade <2%, and no reported cases of constriction.^51^ However, PCIS can still lead to significant morbidity, underscoring the need for ongoing research to refine diagnostic criteria, optimize treatment strategies, and improve patient recovery.

As was noted above, recent advances in imaging, particularly the use of LGE cardiac magnetic resonance (LGE CMR), have significantly improved the management of pericarditis by enabling more precise diagnosis and guiding therapy. LGE CMR allows for the assessment of pericardial inflammation and scarring, helping to tailor treatment intensity and monitor response, which may reduce the risk of complications and recurrence. This represents a paradigm shift in pericarditis care, moving away from empiric treatment approaches toward individualized, imaging-guided management. Importantly, it also facilitates the early identification of patients who may respond to nonsteroidal therapies, thereby helping to avoid unnecessary corticosteroid exposure and its associated risks.

## The new era of pericarditis care

**Figure 6:**
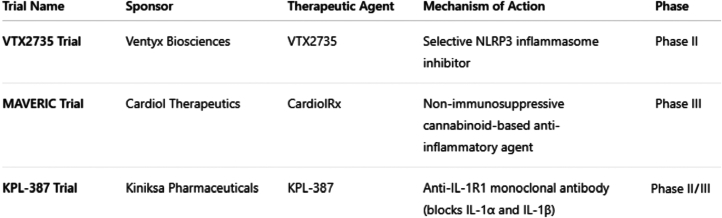
**Ongoing Clinical Trials in Recurrent Pericarditis** Emerging therapies for recurrent pericarditis under investigation in ongoing clinical trials. IL= interleukin.

In light of recent therapeutic advances, the treatment landscape for recurrent pericarditis is undergoing meaningful transformation. Targeted immunomodulatory strategies, once theoretical, are now becoming viable clinical options. The demonstrated efficacy of IL-1R antagonists, specifically anakinra (AIRTRIP [Anakinra-Treatment of Recurrent Idiopathic Pericarditis] randomized clinical trial) and rilonacept (RHAPSODY [Rilonacept Inhibition of Interleukin-1 Alpha and Beta for Recurrent Pericarditis: a Pivotal Symptomatology and Outcomes Study]) in patients with recurrent or corticosteroid-dependent disease, has established a foundation for mechanism-based therapy in select populations.^52^, ^53^, ^54^, ^55^ As research continues to refine our understanding of pericardial inflammation, more precise and accessible interventions are emerging to complement existing approaches. Several clinical trials are currently underway investigating next-generation therapies for recurrent pericarditis, including Ventyx Biosciences' VTX2735 trial, Cardiol Therapeutics' Impact of CardiolRxTM over 6 Months Following IL-1 Blocker Cessation in Pericarditis Patients (MAVERIC) trial, and Kiniksa Pharmaceuticals KPL-387, as outlined in Figure 6.

VTX2735 is an oral, selective inhibitor of the NLRP3 inflammasome, a central regulator of the innate immune response implicated in autoinflammatory conditions.^56^ By attenuating pro-inflammatory mediators such as IL-1β, IL-6, and CRP, without broad immunosuppressive effects, VTX2735 represents a targeted approach that may offer a more tailored alternative for patients with recurrent disease. Ongoing studies will clarify its clinical utility, safety profile, and long-term impact on disease control. The MAVERIC trial, a global Phase III study sponsored by Cardiol Therapeutics, is evaluating CardiolRx, a nonimmunosuppressive oral therapy intended to maintain remission following withdrawal of IL-1 inhibitors.^57^ This multinational trial aims to assess the safety and efficacy of CardiolRx in reducing recurrence rates and sustaining disease control, potentially offering an oral alternative to biologic agents in appropriately selected patients. Adding to this momentum, Kiniksa Pharmaceuticals is advancing the development of KPL-387, a fully human immunoglobulin G2 (IgG2) monoclonal antibody that targets IL-1R type 1, thereby inhibiting the activity of both IL-1α and IL-1β, key drivers of inflammation in recurrent pericarditis.^58^ Designed for monthly subcutaneous administration, KPL-387 is being studied with the goal of offering a specific, durable, and convenient treatment option. By intercepting IL-1 signaling upstream of the inflammatory cascade, KPL-387 may provide sustained remission while reducing the need for frequent dosing.

### Study Limitations

While this review highlights recent advances in the diagnosis and management of PCIS, several important limitations must be acknowledged. First, access to advanced imaging modalities such as CMR remains uneven across health care systems. Despite offering unparalleled tissue characterization, CMR use is often limited by cost, scheduling delays, lack of expertise, and reimbursement issues. In many community hospitals and low-resource settings, CMR may be unavailable altogether, limiting its role in routine PCIS diagnosis and follow-up.

Second, biologic therapies, including IL-1 inhibitors, pose financial and logistical challenges. These agents often require prior authorization, have high out-of-pocket costs, and may not be covered by all insurance plans. The need for cold storage, injections, and frequent follow-up complicates their use. Oral agents under investigation may address some of these barriers, but cost-effectiveness data are lacking, and regulatory approvals remain pending. In many settings, biologics are unavailable or cost-prohibitive, widening global disparities. Scalable, evidence-based treatment algorithms are urgently needed.

Finally, much of the data informing emerging therapies come from small, select populations and may not generalize to broader cohorts or real-world settings. Future studies should include health-economic analyses, assess implementation barriers, and address equity in access across diverse clinical environments.

## Conclusions

Currently available medical therapies and these cutting-edge trials reflect a turning point. The integration of next-generation therapies into the pericarditis treatment paradigm could redefine the standard of care, ushering in an era of greater precision, fewer relapses, and a profoundly improved outlook for patients worldwide. Equally transformative is the growing role of multimodality imaging, with cardiac magnetic resonance imaging at the forefront, offering unparalleled insight into pericardial inflammation and enabling earlier, more accurate diagnosis. This convergence of advanced imaging and novel therapeutics marks a pivotal evolution in the management of pericarditis. The future is actively unfolding, and these innovations are lighting the way forward.

## Funding support and author disclosures

Dr Klein has served on the scientific advisory board of 10.13039/100016492Kiniksa, Cardiol Therapeutics, and Ventyx; and has received research grants from 10.13039/100016492Kiniksa, Cardiol Therapeutics, and Ventyx. All other authors have reported that they have no relationships relevant to the contents of this paper to disclose.
